# Genetic characterisation of staphylococci of food-producing animals in Senegal. PVL detection among MSSA

**DOI:** 10.1186/s12917-019-2137-9

**Published:** 2019-11-04

**Authors:** Olouwafemi Mistourath Mama, Modou Dieng, Bocar Hanne, Laura Ruiz-Ripa, Codou Gueye Mar Diop, Carmen Torres

**Affiliations:** 1 Departamento Agricultura y Alimentación, Área de Bioquímica y Biología Molecular, Universidad de La Rioja, Madre de Dios 51, 26006 Logroño, Spain; 2 LAE/ Ecole Supérieure polytechnique de Dakar, UCAD, Dakar, Sénégal; 3 Service Vétérinaire, Société de gestion des abattoirs du Sénégal, Dakar, Sénégal

## Abstract

**Background:**

Food-producing animals can be a vehicle for staphylococcal species as well as their virulence and antimicrobial resistance genes. This work aimed to analyse the diversity of staphylococcal species in food-producing animals in Dakar/Senegal, and to determine the antimicrobial resistance phenotype/genotype and virulence factors of recovered isolates. Nasal samples of 149 cows and 199 chickens (348 animals) were collected from one slaughterhouse and a local market respectively, and were inoculated on selective media for staphylococci recovery. For *S. aureus* isolates, molecular typing (*spa*-type, MLST) was performed by PCR/sequencing, and the presence of 27 virulence genes (exfoliative and toxic shock toxins, PVL, haemolysins and enterotoxins) as well as the gene *scn* were analysed by PCR. Susceptibility to twelve antibiotics was studied by disc-diffusion method for all staphylococci; the resistance genes involved were screened by PCR.

**Results:**

*Staphylococcus* spp. was present in 3 and 26.8% of chicken and cow nasal samples, respectively. Seven *S. aureus* isolates and forty isolates of other staphylococcal species were identified. *S. aureus* isolates were recovered from cow (*n* = 6) and chicken (*n* = 1) samples, belonging to four genetic lineages: t084/ST15 (*n* = 1); t10579/ST291 (*n* = 3); t355, t4690/ST152 (*n* = 2); and t6618/ST6 (n = 1). All *S. aureus* were methicillin-susceptible, penicillin-resistant (*blaZ*), and two of them were also tetracycline-resistant [*tet*(K)]. All the isolates carried at least one of the virulence genes tested. The PVL genes were detected in three ST15 and ST152 isolates. They all harboured haemolysins encoding genes and lacked the *scn* gene. The other staphylococci recovered were *S. sciuri* (*n* = 16), *S. simulans* (*n* = 11), *S. hyicus* (*n* = 5), *S. haemolyticus* (*n* = 4), *S. chromogenes* (*n* = 3), and *S. hominis* (n = 1); they were all methicillin-susceptible and 27.5% tetracycline-resistant [*tet*(K) and *tet*(L)].

**Conclusions:**

A low prevalence of *S. aureus* was detected among food-producing animals, all susceptible to methicillin. However, the presence of virulence genes (*lukF/lukS-PV*, *eta*, *tst*, *sea* and *see*) is worrisome to the extent that they could be transferred to derived food and therefore, to humans.

## Background

*Staphylococcus* species are common colonizers of skin and mucous membranes of humans and different animal species, but can become opportunistic pathogens causing skin and soft tissues infections (SSTIs) and mastitis, among others [1]. Furthermore, they are current contaminants of animal-derived food, being responsible for food intoxication [2]. *S. aureus* in particularly can express a large variety of pathogenicity factors, such as the staphylococcal enterotoxins, toxic shock syndrome toxin (TSST-1), and Panton-Valentine leucocidin (PVL), among others [3]. In fact, PVL is the most important toxin produced by *S. aureus*; it destroys membranes of host defence cells and erythrocytes by the synergetic action of two specific proteins named LukS-PV and LukF-PV [4]. PVL is though involved in severe skin infections, haemolysis, leucocyte destruction and necrosis [4] . PVL-positive methicillin susceptible *S. aureus* (MSSA) is considered endemic in the African continent [5]. The toxin has been detected worldwide in MSSA and methicillin resistant *S. aureus* (MRSA) isolates of diverse ecosystems, including humans (clinical or community-associated isolates) [6], wildlife [7], farm animals and animal-derived food [8]. The presence of the toxin in such different environments and in MRSA is a concern for public health and food safety, mainly for African regions, especially in those areas in which access to healthcare is limited. Moreover, *S. aureus* strains adapted to humans carry an innate immune evasion cluster (IEC) system that protects them against the human immune system [9]. The IEC consists of several genes, the combination of which gives a determined IEC type. The gene *scn*, since present in all the IEC types, is considered to be a marker-gene for the detection of the IEC [9]. The presence of this system in a *S. aureus* isolate would suggest a human origin.

In general, studies about molecular epidemiology of staphylococci in African countries are being mainly performed in hospital environments [10, 11] and in the food sector [12], but there is scarce data related to farm animals [8]. Furthermore, those studies essentially focus on *S. aureus* species and most of them are performed in the Northern, Central and Southern regions of Africa [8]; consequently, data from West Africa is scarce, especially regarding Senegal. In Senegal, a study was carried out in 2012 on *S. aureus* from pigs and pig farmers, which highlighted the predominance of the clonal complexes CC152 and CC15, the low rate of resistance to methicillin and the frequent detection of PVL toxin [13], and a few years ago a review revealed that CC398, an emergent Livestock-associated *S. aureus* lineage in Europe [2, 14–17], was almost absent in animals and food in Africa [8]. In the said context, this study attempts to provide new information on molecular diversity, antimicrobial resistance and virulence determinants for *S. aureus* and other staphylococcal species in other food-producing animals (such as cow and chicken,) in Senegal (West Africa), and proposes an analysis of the potential occurrence of the lineage CC398 in the area.

## Results

### Staphylococcus species detection

*Staphylococcus* spp. were present in 3 and 26.8% of chicken and cow nasal samples, respectively. *S. aureus* was detected in seven of the tested animals (six cows and one chicken) (Table 1), whereas other staphylococcal species were found in cows (*n* = 35 isolates) and chickens (*n* = 5 isolates) (Table 2). One *Staphylococcus* isolate was obtained from all positive samples, except for one cow sample with two isolates (*S. aureus* and *S. hyicus*). A total of 47 staphylococci were recovered: *S. aureus* (*n* = 7), *S. sciuri* (*n* = 16), *S. simulans* (*n* = 11), *S. hyicus* (*n* = 5), *S. haemolyticus* (*n* = 4), *S. chromogenes* (*n* = 3), and *S. hominis* (n = 1).

**Table 1:** *S. aureus* from nasal samples of healthy cows and chicken: phenotypic and genotypic characteristics

				Antimicrobial resistance			
Origin	Strain	ST/CC	*Spa*-type	Phenotype^a^	Genotype	Virulence genes	*scn*
Cow	C10068	ST6/CC6	t6618	PEN-TET	*bla*Z, *tet*(K)	*sea*, *see*, *hla*, *hlb*, *hld*	negative
Cow	C10067	ST15/CC15	t084	PEN-TET	*bla*Z, *tet*(K)	*eta*, *hla*, *hld*	negative
Cow	C10064	ST291	t10579	PEN	*bla*Z	*tst, hla*, *hlb*, *hld*, *hlg*	negative
Cow	C10066	ST291	t10579	PEN	*bla*Z	*tst, hla*, *hlb*, *hld*, *hlg*	negative
Cow	C10063	ST291	t10579	PEN	*blaZ*	*tst*, *hla*, *hlb*, *hld*, *hlg*	negative
Cow	C10065	ST152/CC152	t4690	PEN	*bla*Z	*lukF/lukS-PV*, *hla*, *hlb*, *hld*, *hlg*	negative
Chicken	C10056	ST152/CC152	t355	Susceptible	–	*lukF/lukS-PV*, *hla*, *hlb*, *hld*, *hlg*	negative

^a^*PEN* penicillin, *TET* tetracycline

**Table 2:** Non-*aureus* staphylococcal species in nasal samples of healthy cows and chicken: phenotypic and genotypic characteristics

		Antimicrobial Resistance	
Origin (n° of positive animals)	Species (n° of isolates)	Phenotype^a^ (n° of isolates)	Genotype (n° of isolates)
Cow (35)	*S. sciuri* (16)	TET (2)	*tet*(K) (2)
		TET (1)	*tet*(K)
		Susceptible (13)	–
	*S. simulans* (8)	TET (2)	*tet*(K) (2)
		Susceptible (6)	–
	*S. haemolyticus* (3)	SXT (1)	*dfrG*
		Susceptible (2)	–
	*S. chromogenes* (3)	Susceptible (3)	
	*S. hyicus* (5)	TET (1)	*tet*(K)
		Susceptible (4)	–
Chicken (5)	*S. simulans* (3)	PEN-TET (1)	*bla*Z, *tet*(K)
		TET-SXT (2)	*tet*(L) (2), *dfrK* (2)
	*S. haemolyticus* (1)	TET-SXT	*tet*(K), *tet*(L), *dfrK*
	*S. hominis* (1)	ERY-TET	*tet*(L), *msr*(A)*/msr*(B)

^a^*PEN* penicillin, *TET* tetracycline, *ERY* erythromycin, *SXT* trimethoprim/sulfamethoxazole

### *Staphylococcus aureus* isolates: antimicrobial resistance, molecular typing and virulence

All seven *S. aureus* isolates were susceptible to cefoxitin and were therefore considered as MSSA. The six isolates of cow origin showed resistance for penicillin (with *blaZ* gene) and two of them also to tetracycline (with *tet*(K) gene). The *S. aureus* isolate of chicken origin showed susceptibility to all antimicrobials tested.

Five *spa*-types were detected among the *S. aureus* isolates, associated with four sequence-types (STs): t084/ST15 (*n* = 1); t10579 /ST291 (*n* = 3); t355, t4690 /ST152 (*n* = 2); and t6618 /ST6 (n = 1) (Table 1). All the isolates were negative for the clonal complex (CC) 398 specific PCR.

The genes encoding for PVL were detected in 2 out of 7 MSSA isolates (28.6%), specifically in t355/ST152 and t4690/ST152 isolates from chicken and cow origins, respectively. The *eta* and *tst* virulence genes were found in isolates of cow origin: 1) *eta* gene in one isolate t084/ST15; and 2) *tst* gene in three t10579/ST291 isolates. The enterotoxin genes *sea* and *see* were present in one isolate of lineage t6618/ST6 recovered of a cow. All the isolates hosted haemolysin encoding genes. In addition, all *S. aureus* isolates lacked the *scn* gene (IEC-negative). This data is summarised in Table 1.

### Non-aureus staphylococci: antimicrobial resistance phenotype and genotype

Among the 40 non-*S. aureus* isolates, 32.5% showed resistance to at least one antimicrobial agent tested. The following resistance rates and genotypes were observed: tetracycline [27.5%; *tet*(K), *tet*(L)], trimethoprim/sulfamethoxazole (SXT) (10%; *dfrG*, *dfrK*), penicillin (2.5%; *blaZ*), erythromycin [2.5%; *msr*(A)*/msr*(B)] and clindamycin (2.5%). It should be noted that resistance to tetracycline was mediated only by *tet*(K) gene for the isolates from cow origin, and by either *tet*(K) or *tet*(L) for the isolates recovered from chicken; furthermore, *dfrG* was present in one isolate from cow, whereas *dfrK* was detected in isolates of chicken origin.

## Discussion

Farm animals are a source for staphylococcal species as well as for their resistance genes and virulence factors [28]. The transmission to humans could occur either through direct contact or via animal derived food [1], hence the importance of analysing staphylococci from food-producing animals.

In this study, the frequency of detection of *S. aureus* was low in cows (4%) and chickens (0.5%). A similar study from Nigeria showed a rate of 2.6% in cattle from slaughterhouses [29]. A higher detection rate was observed in other animals intended for human consumption, such as pigs in Senegal (12.3%) [13] as well as goat and sheep, according to studies carried out in Tunisia [28, 30].

Among the *S. aureus* detected in our work, the most frequently detected lineage was ST291, followed by ST152; however, a similar study performed in Senegal on isolates of pigs and pig farmers showed a predominance of the lineages ST15 and ST152, containing the PVL genes [13]. The sequence type ST291 is a ST398 double locus variant, which encodes two specific subunits, *sauI-hsdS1* and *sauI-hsdS2,* located in GIα and GIβ genomic islands respectively, whereas CC398 isolates encode a single *sauI-hsdS1,* located in GIα [31]. Furthermore, *sauI-hsdS1* of ST291 showed 60% nucleotide similarity to the CC398 *sauI-hsdS1;* consequently, the CC398 specific PCR cannot identify ST291 isolates as part of the CC398 cluster [25, 31], as was the case in our study. The lineage ST291 has been previously described as the major lineage in cattle with mastitis in Egypt [32]; they were all MSSA harbouring *scn* and PVL genes, unlike our isolates. The lineage ST15, mainly associated to MSSA isolates, frequently harbours PVL and enterotoxins [5] and is highly prevalent in African countries, according to the findings of healthcare institutions [33, 34]. Nevertheless, this lineage has also been found in animals (cattle, poultry and donkeys) [3, 8, 30]. The clonal complex CC152 was reported as one of the major clonal complexes in many African countries (healthcare environment) (Madagascar, Morocco, Cameroon, Gabon, Niger, Nigeria, Ghana, Mali and Senegal) [13, 33]. The lineage ST152 is sporadically associated to community-associated (CA) MRSA in some European countries, whereas ST152-MSSA is a particularly frequent clone in Western and Central Africa [33, 35]. PVL is the most important toxin secreted by *S. aureus* and is involved in severe skin infections and life-threatening diseases. This toxin is found all over the world, mainly among CA *S. aureus* isolates [6, 35]. Nonetheless, it was also described in isolates from farm/wild animals (linked to the lineages ST5, ST8, ST15, ST80, ST152, and ST121) [36–38] and animal-derived food (linked to the lineages ST8, ST121, and ST152) [3, 39]. PVL is very frequently harboured by MSSA isolates in Africa, where PVL-positive *S. aureus* is considered endemic [5]. In this work, all the PVL-positive isolates were MSSA (28.6% of *S. aureus* detected), contrary to the results obtained from healthy sheep in Tunisia, showing only PVL-positive MRSA (6.8% of *S. aureus* detected) [40]. In a previous study performed on pigs in Senegal, 38.4% of the *S. aureus* isolates harboured the PVL toxin, being 78.6% of them MSSA [13]. Contrarily to the above mentioned studies highlighting the recurrence of PVL-positive *S. aureus* among animals intended for human consumption in Africa, the absence of PVL was noted among *S. aureus* recovered from donkeys for meat consumption in Tunisia [30]. Furthermore, other virulence encoding genes were detected among the isolates (*sea*, *see*, *eta*, *tst*, *hla*, *hlb*, *hld* and *hlg*). The presence of staphylococcal enterotoxins (SEs) in bovine isolates is worrisome since, as the literature shows, SEs are detected more often in cows with mastitis than in healthy cows [41]. Furthermore, SEA is the enterotoxin most frequently reported in food (encoded by *sea* gene) and the main cause of staphylococcal food poisoning (SFP) in many countries [42]; it is generally detected in meat, poultry and milk, among others. Nevertheless, SEE (encoded by *see* gene) is rarely reported in food and food-producing animals, although it was involved in some cases of SFP outbreaks in France [42]. Interestingly, none of our isolates carried the genes which encode the toxins SEC or SED, described as the most recurrent in bovines [43]. The presence of such virulent factors in *S. aureus* from food-producing animals, especially in African countries like Senegal, is a big concern for public health to the extent that in some cases, the animals are raised in the houses or sold in open markets, where they are in contact with people and retail food products. This easily results in the dissemination of staphylococcal virulence genes in different niches of the community. The lack of *scn* gene in our isolates suggests their being of animal origin, as expected, thus discarding a potential human origin (by handlers during slaughter).

In addition, other species were detected, the most prevalent being *S. sciuri* and *S. simulans,* followed by *S. hyicus*, *S. haemolyticus*, *S. chromogenes* and *S. hominis*. The coagulase-negative species mentioned above seem to be frequent in cattle and poultry samples [36, 44, 45]. Increasingly considered as opportunistic pathogens for humans and animals [44], coagulase-negative staphylococci are thought to be a reservoir for important resistance genes that could be transferred to *S. aureus* isolates [45], hence the importance of their surveillance.

Regarding the antimicrobial resistance, the phenotypes and genotypes observed in this study are frequent among *S. aureus* isolates from food-producing animals and animal-derived food [12, 28, 36, 38]. Similar phenotypes were previously observed among pig isolates of the same country [13]. Resistance to at least one antimicrobial agent was evidenced in 32.5% of the non-*S. aureus* isolates tested (tetracycline, SXT, penicillin, erythromycin and clindamycin), maybe due to the very frequent use of beta-lactams, tetracyclines, lincosamides and sulphonamides in the veterinary sector (food-producing animals and pets) [46].

## Conclusion

A relatively low prevalence of *S. aureus* has been observed in nasal samples of food-producing animals (chickens and cows) in Senegal, with *S. aureus* being MSSA in all cases. Nevertheless, all the *S. aureus* isolates detected harboured at least one virulence gene (especially PVL and enterotoxins genes), which could be a concern for food-safety and public health, particularly in a developing country with areas in which access to medical care is difficult and limited.

## Methods

### Sample collection

From May to July 2017, nasal samples of 149 cows and 199 chickens (348 animals) were taken with aseptic swabs in the General Society of Slaughterhouses of Senegal (SOGAS) and a local market, respectively. In all cases, nasal samples were obtained from dead animals, just after they were sacrificed to human consumption as part of routine work in the slaughterhouse and the market.

### Isolation and identification of staphylococci strains

Tubes of 5 ml of Brain Heart Infusion (BHI) broth (+NaCl 6.5%) were inoculated with the nasal swabs and then incubated at 37 °C for 24 h. After growth, bacterial culture was distributed on plates of mannitol-salt-agar (Conda, Madrid/Spain), Baird Parker (Becton Dickinson, Heidelberg/Germany) and oxacillin-resistance-screening-agar-base (Oxoid, Hampshire/England) for *S. aureus* and MRSA recovery. Non-*aureus* staphylococci were also identified and characterised. Up to two colonies/plate with staphylococcal morphology were isolated and subjected to Dnase agar test (Conda, Madrid/Spain) and identification by matrix-assisted laser desorption/ionization time of flight (MALDI-TOF) mass spectrometry (Bruker, Massachusetts/USA).

### Antibiotic susceptibility and resistance genes detection

For all staphylococci identified, susceptibility to penicillin (10 units), cefoxitin (30 μg), gentamicin (10 μg), tobramycin (10 μg), tetracycline (30 μg), chloramphenicol (30 μg), erythromycin (15 μg), clindamycin (2 μg), ciprofloxacin (5 μg), linezolid (30) and SXT (1.25 + 23.75 μg) was analysed by disk-diffusion method [18]. In addition, susceptibility to streptomycin (30 μg) was also tested [19]. Antimicrobial resistance genes were determined by PCR, according to the resistance phenotype of the isolates: penicillin (*blaZ*)*,* tetracycline [*tet*(K)*, tet*(L)*,* and *tet*(M)]*,* macrolides [*erm*(A), *erm*(B), *erm*(C), *msr*(A)*/msr*(B)], and trimethoprim (*dfrA*, *dfrD*, *dfrG*, *dfrK*) [20–23] .

### Molecular typing and virulence genes study in *S. aureus* isolates

*Spa*-typing and Multilocus sequence typing (MLST) were performed for *S. aureus* strains by polymerase chain reaction (PCR) and sequencing and the *spa*-type, sequence type (ST), and clonal complex (CC) were determined as previously described [24]. In addition, a specific PCR was performed for the livestock-associated CC398 lineage detection [25]. The presence of the genes encoding the PVL (*lukF/lukS-PV*), exfoliative toxins (*eta* and *etb*), toxic shock syndrome toxin (*tst*), haemolysins (*hla*, *hlb*, *hld*, *hlg* and *hlgv*) and SEs (*sea*, *seb*, *sec*, *sed*, *see*, *seg*, *seh*, *sei*, *sej*, *sek*, *sel*, *sem*, *sen*, *seo*, *sep*, *seq*, *ser*, and *seu*) was screened by PCR [14, 26, 27]. The gene *scn,* was also tested for *S. aureus* isolates [14]. Positive and negative control strains of the University of La Rioja were included in all PCR reactions.

## Data Availability

The datasets used and/or analysed during the current study are available from the corresponding author on reasonable request.

## Abbreviations

BHI: Brain Heart Infusion
CA: Community-Associated
CC: Clonal Complex
IEC: Immune Evasion Cluster
MALDI-TOF: Matrix-Assisted Laser Desorption/Ionization Time Of Flight
MLST: Multilocus Sequence Typing
MRSA: Methicillin-Resistant *S. aureus*
MSSA: Methicillin-Susceptible *S. aureus*
PCR: Polymerase Chain Reaction
PVL: Panton-Valentine Leucocidin
SEs: Staphylococcal Enterotoxins
SFP: Staphylococcal Food Poisonning
SSTIs: Skin and Soft Tissues Infections
ST: Sequence Type
SXT: Trimethoprim/Sulfamethoxazole
TSST: Toxic Shock Syndrome Toxin
